# Systemic Inflammation-Related Bradycardia in COVID-19

**DOI:** 10.1155/2021/9986955

**Published:** 2021-09-14

**Authors:** Aswin Srinivasan, Tusharkumar Pansuriya, Branden Wilson, Siva T. Sarva, Turuvekere Jayaram, Salman Alim, Ramesh Kesavan, Syed Raza

**Affiliations:** ^1^Department of Internal Medicine, HCA Houston Kingwood/University of Houston College of Medicine, USA; ^2^Department of Pulmonology/Critical Care, HCA Houston Kingwood/University of Houston College of Medicine, USA; ^3^Department of Cardiology, HCA Houston Kingwood/University of Houston College of Medicine, USA

## Abstract

Systemic inflammation-related sinus bradycardia in COVID-19 infection has not been well described yet. This six-patient case series excludes common causes of bradycardia. As bradycardia may be a sequela of COVID-19 infection, we recommend closely monitoring hemodynamics and stopping medications that can exacerbate bradycardia in these patients.

## 1. Introduction

Coronavirus disease 2019 (COVID-19), caused by the pathogen SARS-CoV-2, is primarily a respiratory illness with secondary systemic and cardiovascular involvement reported [1]. Acute myocardial injury is the most commonly reported cardiac manifestation, with elevations in serum troponin reported in 8-10% of patients and troponin elevations associated with increased mortality [2]. Additionally, it has been reported that COVID-19 patients with hyperglycemia, even without known diabetes, have higher risk of severe disease than those with normoglycemia [3]. This issue in COVID-19 patients is important as tight glycemic control has been described as affecting cardiovascular outcome [4]. Arrhythmias have also been noted, with a recent report from Wuhan, China, noting that 16.4% of hospitalized patients and 44% of ICU patients developed arrhythmias [1]. This phenomenon was also reported in a global survey of electrophysiologists conducted by the Heart Rhythm Society, which reported that bradyarrhythmias occurred in conjunction with COVID-19 [2]. In this case series, we report a new arrhythmic phenomenon of sinus bradycardia without underlying myocardial injury in six patients with COVID-19. We recommend close monitoring of hemodynamic stability and stopping medications that can further exacerbate bradycardia in these patients.

## 2. Case Description

**Figure 1:**
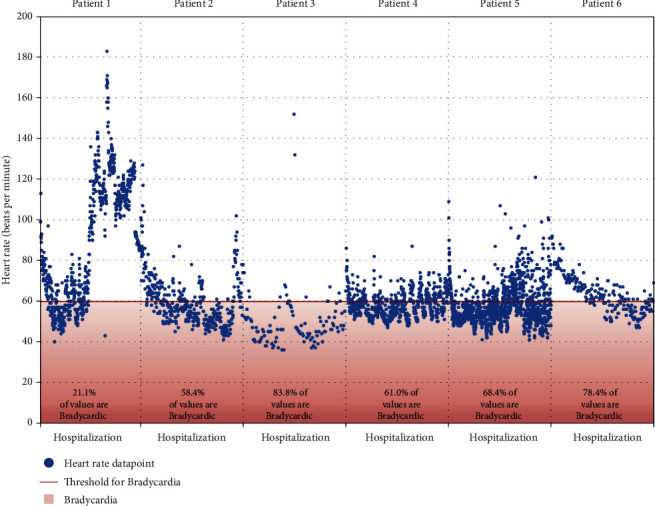
Heart rate throughout hospitalization for each patient. Every discrete heart rate datapoint as recorded in the electronic medical record throughout each patient's hospital stay, with patients' data shown adjacent to one another using a uniform *y*-axis, but individually labeled *x*-axes. The proportion of recorded values found to be bradycardic is noted at the bottom of each patient's dataset. HR: heart rate; %: percentage; *x*-axis: duration of hospitalization (continuous measure of time, no discrete intervals used); *y*-axis: heart rate in beats per minute.

**Figure 2:**
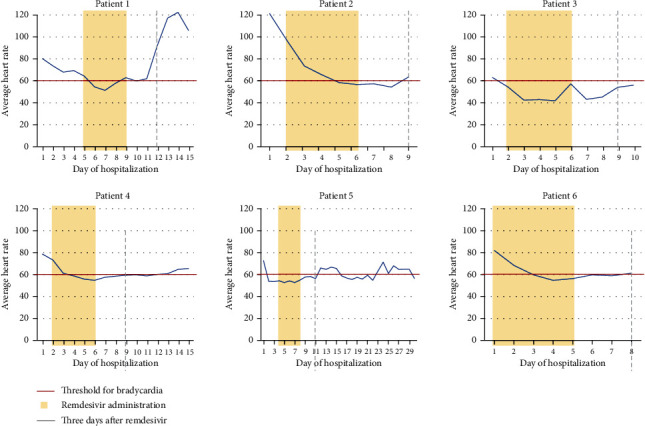
Average heart rate throughout hospitalization juxtaposed with remdesivir administration. Each patient's average daily heart rate in beats per minute is juxtaposed in this figure with documented hospital days of remdesivir administration. Every patient received five consecutive doses of remdesivir, starting on the first full day of hospitalization unless otherwise noted in the case descriptions. The second documented day of hospitalization was the first full day of hospitalization for patients who were admitted in the afternoon or evening of the day prior. The first day and last days of administration as follows in a “first day/last day” format: patient (1) 5/9, patient (2) 2/6, patient (3) 2/6, patient (4) 2/6, patient (5) 4/8, patient (6) 1/5. The threshold for bradycardia is noted, and the third day after the last dose of Remdesivir is noted. Recovery from bradycardia was expected at this point should remdesivir be causative.

Patient 1 presented with weakness, dyspnea, and dizziness since testing positive for SARS-CoV-2 five days before admission. His admission electrocardiogram (EKG) and laboratory studies were unremarkable (Table 1). He required high-flow nasal cannula (HFNC). Chest X-ray (CXR) showed pulmonary infiltrates consistent with bilateral pneumonia. Given his worsening respiratory status, convalescent plasma, dexamethasone, and full-dose enoxaparin were started. He experienced persistent bradycardia for five days, starting on day six of his hospitalization (Figures 1, 2). His oxygen requirements progressively increased, with a CXR on hospital day eleven showing worsening moderate bilateral pneumothorax, for which a thoracostomy was performed. Despite treatment, the patient became hypothermic, tachypneic, hypotensive, and eventually passed away from pulseless electrical activity.

Patient 2 presented with worsening shortness of breath (SOB) after testing positive for SARS-CoV-2 five days before admission. Her admission EKG and laboratory results were unremarkable (Table 1). She required a nonrebreather and BiPAP. CXR revealed extensive bilateral pulmonary infiltrates. Dexamethasone, ceftriaxone, azithromycin, and full-dose enoxaparin were initiated. Remdesivir and convalescent plasma initiated when her respiratory status worsened. She developed bradycardia on hospital day four, which resolved on day 9 (Figures 1 and 2). Once her respiratory status improved and oxygen requirements decreased, she was discharged home with dexamethasone.

Patient 3 presented with ten days of worsening SOB, dry cough, fever, and generalized body pain. She tested positive for SARS-CoV-2 four days before admission. CXR revealed extensive bilateral pulmonary infiltrates. Though she required HFNC, her admission EKG and laboratory studies were unremarkable. Dexamethasone, remdesivir, convalescent plasma, and azithromycin were initiated. On hospital day three, she developed eight days of persistent bradycardia (Figures 1 and 2). Once her respiratory status improved, oxygen requirement decreased, and heart rate improved, she was discharged with prednisone.

Patient 4 presented with SOB and cough after testing positive for SARS-CoV-2 nine days before admission. CXR revealed bilateral pneumonia. Though she required HFNC, her admission EKG and laboratory studies were unremarkable. Remdesivir, convalescent plasma, azithromycin, dexamethasone, and full-dose anticoagulation were started on admission. She started experiencing persistent bradycardia lasting eight days on hospital day four (Figures 1 and 2). Once her respiratory status and heart rate improved, she was discharged.

Patient 5 presented with SOB after testing positive for SARS-CoV-2 seven days before admission and being discharged from the ED on steroids. His admission EKG and laboratory studies were only remarkable for elevated liver function tests; he now required HFNC (Table 1). CT of the chest showed ground glass opacities. He received convalescent plasma, dexamethasone, and full-dose enoxaparin, with remdesivir initiation on hospital day three after improvement in kidney and liver function (Tables 1 and 2). He developed sinus bradycardia on the same day. Once his respiratory status improved, he was discharged on albuterol and dexamethasone.

Patient 6 presented with SOB after testing positive for COVID-19 infection seven days before admission. His admission EKG and laboratory studies were unremarkable, although he required HFNC and subsequently BiPAP (Table 1). CXR showed bilateral pneumonia. He received convalescent plasma, remdesivir, dexamethasone, cefepime, and full-dose enoxaparin. On hospital day three, he developed persistent bradycardia lasting five days (Figures 1 and 2). Once his respiratory status and oxygen requirement improved, he was discharged with dexamethasone and amlodipine.

## 3. Discussion

Cardiac phenomena associated with COVID-19 include direct myocardial injury, myocarditis, heart failure, and cardiogenic shock [1]. However, COVID-19-associated arrhythmias are not well described in the literature. In this case series, we present several patients with sinus bradycardia associated with COVID-19 in the absence of myocardial injury.

Our case series includes six patients—three male and three female. None had any reported history of coronary artery disease. Five patients had a prior history of hypertension. Echocardiography was unremarkable except a mildly reduced ejection fraction of 45-50% in patient 5. On admission, every patient exhibited normal sinus rhythm on EKG, with patients developing sinus bradycardia while hospitalized despite their generally healthy baseline cardiac status (Table 1). The lowest daytime heart rates for every patient were 46, 43, 36, 48, 42, and 35 beats/min, respectively. Severity of bradycardia that each patient exhibits does not appear to be related to their cardiovascular morbidity.

On admission, EKG on all patients revealed normal sinus rhythm. A retrospective cohort study from Wuhan, China, showed an increase in cardiac troponin I in fatal cases that started around 16 days into their illness [2]. All the six patients in this series had serial troponin enzymes of less than 0.012 during bradycardia episodes, which indicates that sinus bradycardia was not associated with myocardial injury. Thyroid function was within normal limits for all patients. In patients 3 and 4, home thyroid medications were resumed on admission and thyroid function was normal during hospitalization. All patients had a normal range of potassium levels during the bradycardia period. All patients did not receive any AV nodal blocking agents or sedatives prior to or during the period of bradycardia. The patients were not given any AV nodal blocking agents or sedatives prior or during their periods of bradycardia (Table 1).

Recent literature shows remdesivir to be a potential cause of sinus bradycardia [5]. But patient 5 developed bradycardia prior to administration of remdesivir. The half-life of remdesivir is approximately one hour, and the half-life of its active metabolite is approximately 27 hours. However, patients 2, 3, and 4 did not recover to normal sinus rhythm even after three to four days of the last dose of remdesivir administration (Figure 2). This finding indicates remdesivir likely is not the sole cause of bradycardia.

Another virus in the same family, severe acute respiratory syndrome coronavirus (SARS-CoV), has been found to resemble closely with SARS-CoV-2 [6]. Interestingly, sinus bradycardia has been previously reported in 18 patients (14.1%) with SARS-CoV [6]. It is possible that the SARS-CoV-2 and related viruses may be associated with bradycardia secondary to structural interactions at a molecular level between these pathogens and their biological hosts.

Possible mechanisms for bradycardia such as direct myocardial injury, hypoxia, enhanced inflammatory stages, and drug toxicity have been postulated in recent reports [1]. In a multicenter autopsy study performed on 21 patients with SARS-CoV-2, 18 cases showed macrophage interstitial infiltration in the myocardium, while the other 3 cases showed lymphocytic myocarditis [7]. These high levels of myocardial macrophages in COVID-19 patients may largely result from systemic elevations of proinflammatory cytokines such as interleukin-6 (IL-6). Four of the patients in this case series had elevated IL-6 levels (Table 2) at the onset of bradycardia. All six patients exhibited elevated CRP at the onset of bradycardia. The inflammatory cytokines including IL-6 released during the host inflammatory response phase could act on sinoatrial (SA) node. This finding suggests a possible association between bradycardia and host inflammatory response. Finally, the age range of the six patients in this series was 41 and 67 years. The age range of nearly 30 years makes this observation limited to potential bias. Therefore, more studies are needed to investigate and establish the correlation between bradycardia and COVID-19 infection.

## 4. Conclusion

As per our knowledge and published data, association of sinus bradycardia in COVID-19 patients has not been well described yet. In this case series, we excluded all major causes of bradycardia. We recommend closely monitoring these patients for hemodynamic stability, especially the patients with underlying cardiovascular conditions. As described, arrhythmia gets better along with improvement in clinical condition in most cases. We also believe it is important to exclude other main causes of bradycardia and to know that sinus bradycardia is a potential sequela of COVID-19 infection. We recommend to be prudent about not taking any medications that can exacerbate the severe bradycardia further. More studies need to be done in survivors for long-term cardiac sequelae.

## Figures and Tables

**Table 1:** Patient characteristics and data.

Patient	Age (year)	Sex	Relevant past medical history	Initial clinical presentation	Relevant laboratory results on admission	Findings on admission EKG	Days between COVID-19 diagnosis and onset of persistent bradycardia	Findings on EKG(s) after onset of persistent bradycardia
1	45	Male	Hypertension	Weakness, shortness of breath, and dizziness	Troponin: <0.012 Potassium: 3.6 Creatinine: 0.7 TSH: 0.205	Sinus tachycardia; intervals within normal limits; no conduction system blocks	10 days	Hospital day 4: Sinus bradycardia; intervals within normal limits; no conduction system blocks
2	34	Female	Type II diabetes mellitus	Shortness of breath	Troponin: <0.012 Potassium: 4.0 Creatinine: 0.8 TSH: 0.515	Sinus tachycardia; intervals within normal limits; no conduction system blocks	9 days	Hospital day 8: Sinus bradycardia; intervals within normal limits; no conduction system blocks
3	60	Female	Hypertension, hypothyroidism, type II diabetes mellitus	Shortness of breath, cough, fever, myalgias	Troponin: <0.012 Potassium: 4.2 Creatinine: 0.7 TSH: 0.667	Normal sinus rhythm; intervals within normal limits; no conduction system blocks	6 days	Hospital days 3, 4, & 6: Sinus bradycardia; intervals within normal limits; no conduction system blocks
4	67	Female	Hypertension, hypothyroidism, deep vein thrombosis, anxiety disorder	Shortness of breath, cough, fever	Troponin: <0.012 Potassium: 4.1 Creatinine: 0.5 TSH: 0.139	Normal sinus rhythm; intervals within normal limits; no conduction system blocks	12 days	Hospital day 6: Sinus bradycardia; intervals within normal limits; no conduction system blocks
5	54	Male	Hypertension, obstructive sleep apnea	Shortness of breath	Troponin: <0.012 Potassium: 4.1 Creatinine: 0.5 TSH: 0.508	Normal sinus rhythm; intervals within normal limits; no conduction system blocks	8 days	Hospital day 10: Sinus bradycardia; intervals within normal limits; no conduction system blocks Hospital day 15: Sinus bradycardia; prolonged QT/QTc interval (482/477 milliseconds); no conduction system blocks
6	41	Male	Hypertension, obesity	Shortness of breath	Troponin: <0.012 Potassium: 4.9 Creatinine: 0.6 TSH: NA	Normal sinus rhythm; intervals within normal limits; no conduction system blocks	9 days	Hospital day 4: Sinus bradycardia; intervals within normal limits; right bundle branch block, left posterior fascicular block Hospital day 8 (discharge day): Sinus bradycardia; intervals within normal limits; no conduction system blocks

**Table 2:** Additional patient data.

Patient	Lowest HR between 8 AM and 5 PM (bpm)	Peak D-dimer (ng/mL)	Peak CRP (normal range: 0-12.4 mg/L)	IL-6 (normal range: 0-14.6 pg/mL)
1	46	1843	181.4	20.0
2	43	294	167.7	108.9
3	36	831	67.4	NA
4	48	1240	48.1	NA
5	42	41352	201.2	93.6
6	35	878	179.8	125.7

bpm: beats per minute; CRP: C-reactive protein; HR: heart rate; IL-6: interleukin 6; L: liter; mL: milliliter; mg: milligrams; NA: not available; ng: nanograms; pg: picograms.

## Data Availability

Data used to support the findings of this study are available from the corresponding author upon request.

## Abbreviations

COVID-19: Coronavirus disease 2019
EKG: Electrocardiogram
HFNC: High-flow nasal cannula
CXR: Chest X-ray
SOB: Shortness of breath
SARS-CoV: Severe acute respiratory syndrome coronavirus
IL-6: Interleukin-6
CRP: C-reactive protein
SA: Sinoatrial node.

## Additional Points

*Learning Objectives*. (i) To recognize bradycardia as a sequela of COVID-19 infection. (ii) Needs close monitoring of these patients for hemodynamic instability.
